# Overcoming the Refractory Expression of Secreted Recombinant Proteins in Mammalian Cells through Modification of the Signal Peptide and Adjacent Amino Acids

**DOI:** 10.1371/journal.pone.0155340

**Published:** 2016-05-19

**Authors:** Gülin Güler-Gane, Sara Kidd, Sudharsan Sridharan, Tristan J. Vaughan, Trevor C. I. Wilkinson, Natalie J. Tigue

**Affiliations:** Department of Antibody Discovery and Protein Engineering, MedImmune Ltd., Granta Park, Cambridge, United Kingdom; Centro Nacional de Biotecnologia - CSIC / CIF Q2818002D, SPAIN

## Abstract

The expression and subsequent purification of mammalian recombinant proteins is of critical importance to many areas of biological science. To maintain the appropriate tertiary structure and post-translational modifications of such proteins, transient mammalian expression systems are often adopted. The successful utilisation of these systems is, however, not always forthcoming and some recombinant proteins prove refractory to expression in mammalian hosts. In this study we focussed on the role of different N-terminal signal peptides and residues immediately downstream, in influencing the level of secreted recombinant protein obtained from suspension HEK293 cells. Using secreted alkaline phosphatase (SEAP) as a model protein, we identified that the +1/+2 downstream residues flanking a heterologous signal peptide significantly affect secreted levels. By incorporating these findings we conducted a comparison of different signal peptide sequences and identified the most productive as secrecon, a computationally-designed sequence. Importantly, in the context of the secrecon signal peptide and SEAP, we also demonstrated a clear preference for specific amino acid residues at the +1 position (e.g. alanine), and a detrimental effect of others (cysteine, proline, tyrosine and glutamine). When proteins that naturally contain these “undesirable” residues at the +1 position were expressed with their native signal peptide, the heterologous secrecon signal peptide, or secrecon with an additional alanine at the +1 or +1 and +2 position, the level of expression differed significantly and in an unpredictable manner. For each protein, however, at least one of the panel of signal peptide/adjacent amino acid combinations enabled successful recombinant expression. In this study, we highlight the important interplay between a signal peptide and its adjacent amino acids in enabling protein expression, and we describe a strategy that could enable recombinant proteins that have so far proved refractory to expression in HEK293 cells, to be produced in sufficient quantities to answer important biological questions.

## Introduction

The ability to recombinantly express and purify a protein of interest of sufficient quantity and quality is critical for many areas of biological science, not least in the production of biopharmaceuticals and recombinant antigens for therapeutic antibody generation. In the latter case, the target antigen needs to be available in a form that most closely resembles the native protein. Prokaryotic expression hosts such as *E*. *coli* provide a convenient and cost-effective means for producing recombinant proteins, however, the proteins obtained via these systems are not glycosylated and there is a risk that they will not be folded properly [1]. Due to these undesirable properties, mammalian cells such as HEK293, NS0 and CHO are frequently utilised as transient expression hosts. Certain proteins, however, appear to be refractory to transient expression in mammalian hosts, and significant optimisation is required to obtain sufficient material for downstream applications. Several approaches to enable expression or increase the level of expression of such proteins have been adopted including genetic modification of the host cell [1], altering growth parameters, media components [2] and transfection conditions [3], incorporating different promoters [4], codon optimisation of the gene sequence [5], using peptide or fusion protein tags (for example GST, MBP, NusA, Fc) and changing the location of these [6]. In some cases, the generation of stable cell lines, and/or scale-up [1] is required to generate the desired amount of recombinant protein; activities which have significant time and resource implications. In this study we sought to identify a universal strategy to improve the expression of secreted recombinant proteins in HEK293 cells, by investigating the role of N-terminal signal peptides.

During the translation of secreted proteins, the signal peptide is recognised as it emerges from the ribosome; it is bound by the signal recognition particle (SRP) and translation is halted. This entire complex is transported to the external face of the ER where it binds to the SRP receptor, and the signal sequence is transferred to a translocon. Whilst bound to the translocon, translation is reinitiated and the protein passes through the ER membrane and into the lumen. As it does this, the signal peptide is recognised by a signal peptidase and is cleaved to generate the mature protein that can be trafficked through the Golgi network before being secreted from the cell via the classical secretory pathway [7].

Signal peptide sequences were first described in 1975 as short removable N-terminal sequences that mark a protein for removal from the cell [8]. Subsequently, it was found that although these peptide sequences are highly diverse both in length and in amino acid composition, and are found in both prokaryotes and eukaryotes, they share a conserved tripartite structure [9,10]. Each signal peptide (~20–30 residues in length) comprises a basic “N domain”, a ~7–13 residue hydrophobic “H domain” and a slightly polar “C domain” [7]. In addition to this common structure, conservation of residues in particular positions in the mammalian signal peptide sequence has emerged. For example, small neutral amino acids are prevalent in positions -1 (the last amino acid of the signal peptide) and -3, whereas bulky aromatic amino acids are often found at the -2 position. Interestingly, proline residues are virtually absent from all positions from -3 to +1 (first amino acid of the mature peptide) [10]. The highly diverse primary sequence of these signal peptides, however, does suggest that the endogenous signal peptide plays a role in regulating the physiological level of secretion of a particular protein from the cell, and that by replacing the signal peptide the level of expression of the protein can be modified. Indeed, this has shown to be the case in a number of studies, where replacement of the native signal peptides with those from proteins that are known to secrete at high levels (e.g. luciferase, tissue plasminogen activator) can significantly improve secreted levels [11–13].

The potential posed by utilising heterologous signal peptides from highly secreted proteins to improve protein yields is an attractive one and the identification of a universally applicable strategy involving the use of a single signal peptide for all recombinant proteins would constitute an ideal scenario. In an attempt to investigate this potential further, we chose to study the expression of a widely used model secreted protein, secreted alkaline phosphatase (SEAP), but in the context of non-native signal peptides. In this study we identify an optimal signal peptide for the secretion of SEAP, but importantly we also identify a critical role for amino acids in the mature protein (positions +1 and +2). We identify amino acids that negatively influence the expression of SEAP when inserted at the +1 position and by investigating the expression of proteins with unfavourable native +1 amino acids we demonstrate that refractory expression can be restored by replacing the signal peptide or by also placing small neutral amino acids at the +1/+2 positions. It is anticipated that these findings could be applied widely to improve the production of proteins of significant importance to many areas of biological research as well as the biopharmaceutical industry.

## Materials and methods

### Generation of the consensus signal sequence

Signal peptide sequences of 16 amino acids (aa) in length from human proteins were obtained from the Signal Peptide Database (http://www.signalpeptide.de/). A total of 24 sequences were obtained and the frequencies of residues at each position were calculated (S1 Fig). To generate the “consensus” sequence (MLLLLLLLLLLALALA) the most frequent residue at each position was utilised.

### Generation of expression vectors

The expression vector pDest12.2oriP_CD33_FLAG10His, based on the pDest12.2 vector (Thermo Fisher), was generated via gene synthesis and sub-cloning to encode a human CD33 signal peptide, followed by a *Not*I site and an *Nhe*I site, preceding a FLAG tag and 10xhistidine tag. DNA encoding the SEAP gene was optimised for expression in human cells (Geneart, Thermo Fisher) and sub-cloned into this vector. The resultant pDest12.2oriP_CD33_SEAP_FLAG10His vector formed the template for the construction of all further SEAP-containing expression vectors, whereby signal sequences and adjacent residues were replaced by equivalent versions (i.e. using the same codon usage where amino acids were unchanged) using a restriction site upstream of the start codon, and an internal restriction site in the SEAP-encoding sequence. Expression vectors for interferon alpha 2a (IFNα2), interleukin 25 (IL-25), sclerostin, mimecan and prostaglandin-H2 D-isomerase were generated entirely by gene synthesis, incorporating the different signal peptide/adjacent residue combinations.

### Protein expression

All proteins were expressed in a suspension-adapted HEK293 cell line (ExPi293F, Thermo Fisher). Cells, growing in deep 96-well blocks, were transfected in triplicate at a density of 2.5x10^6^/ml using ExpiFectamine™ 293 according to the manufacturer’s instructions. Following transfection, the cells were grown at 37°C by shaking at 350 rpm in 5%CO_2_, 70% humidity for six days. The conditioned media, containing secreted protein, were harvested on day 6 by centrifugation at 3500rpm for 20 minutes. The clarified media from each of the three replicates for each expression vector were pooled and stored at -20°C. Each transfection was performed in duplicate, on separate days, and the conditioned media was analysed separately by Western blot analysis and a SEAP activity assay.

### Western Blot Analysis

An equal volume of each pooled conditioned media sample was incubated in a final concentration of 1 mM DTT and 1xLDS NuPAGE LDS sample buffer (Thermo Fisher) at 70°C for 15 minutes before loading onto a 4–12% Bis-Tris NuPAGE gel (Thermo Fisher), alongside a protein molecular weight marker (MagicMark™ XP Western Protein Standard, Thermo Fisher). The gels were run at 150V, 400 mAmp for 100 minutes in MOPS buffer and the proteins were subsequently transferred onto PVDF membranes using iBlot transfer stacks (Thermo Fisher). The membranes were blocked at 4°C overnight in Odyssey-PBS blocking buffer, followed by an hour with mouse anti-His antibody (MAB050, RnD systems) and a further hour with IRDye 800 CW donkey anti-mouse antibody at ambient temperature. Between each antibody incubation, the membranes were washed five times with Odyssey-PBS blocking buffer containing 0.1% Tween 20 and finally the membranes were rinsed with PBS and left to dry in the dark. The membranes were scanned using the Odyssey scanner (LiCor) at 800nm to enable visualisation of protein bands.

### Protein purification

Purification of His-tagged proteins from the conditioned media of HEK293 cells was performed using a high-throughput 24-well filter plate method. A 500ul sample of conditioned media was applied to a 25ul packed bed of HisTrap Excel resin (GE Healthcare) that had been equilibrated with 2xD-PBS/10mM imidazole. The sample was incubated for 90 minutes with the resin prior to a 25mM imidazole wash step, and elution with 400mM imidazole. Buffer exchange was then performed using a 7,000 Da cut off Zeba desalting spin column (Thermo Scientific) into 2 x DPBS buffer.

#### SEAP activity assay

The amount of SEAP present in the conditioned media of HEK cells transiently transfected with the different SEAP constructs was determined using the Great EscAPe™ SEAP Chemiluminescence Kit 2.0 (Clontech, Takara), accordingly to the manufacturer’s instructions. A standard curve, using purified SEAP was generated and a linear regression analysis performed. All samples were diluted 1000-fold and analysed in replicate (6). The amount of SEAP was calculated using the linear regression equation from the standard curve, and the percentage change to the control sample (Native-SEAP) in each case, was determined. Statistical analyses of each experiment were performed using a one-way ANOVA (Dunnett’s correction for multiple comparisons), and graphs combining the data from the two independent experiments were generated.

### N-terminal sequencing

To enable N-terminal sequencing of proteins, the conditioned media was subjected to Nickel chelate chromatography as described above. The purified proteins were mixed with an equal volume of a reducing sample buffer, subjected to SDS-PAGE electrophoresis, transferred on 0.22 μm Immobillon PSQ PVDF membrane (Millipore) and stained with Coomassie Blue. The main band from each sample was cut out from the membrane and used for sequencing via chemical Edman degradation of the N-terminus. Analysis was performed using an automatic sequencer PPSQ-31A (Shimadzu, Japan).

### IFNα2 expression and purification

IFNα2 was transiently expressed in HEK293F cells, as described above, in 50 ml Erlenmeyer flasks and the conditioned media was harvested after 4 days of expression. The supernatant was diluted two-fold in 2 x DPBS, loaded onto 5 ml His Trap column (GE Healthcare), washed with 40 mM Imidazole and eluted with 400 mM Imidazole. The sample was concentrated using a 5K cut-off Vivaflow 200 concentrator (VWR) and loaded onto a Superose 12 size exclusion chromatography column (GE healthcare) that had been equilibrated with 2 x DPBS buffer. Fractions containing the purified protein were collected and pooled.

### IFNα2 activity

Activity of purified human IFNα2 was measured using iLite^TM^ Type I IFN Assay Ready cells (EuroDiagnostica) that express firefly luciferase under the control of an IFNα/β response element. Cells were incubated with a three-fold serial dilution of purified IFNα2 (four replicates per sample) for 18 hours at 37°C with 5% CO_2_ and the luminescence was measured following addition of a luciferase substrate (Bright-Glo^TM^, Promega). The concentration of IFNα2 required for a half-maximal (EC50) response was determined using non-linear regression analysis (log [agonist] vs. response, 3-parameter fit curve) in GraphPad Prism (San Diego, CA).

## Results

### Impact of the CD33 signal peptide and additional +1/+2 amino acids on SEAP expression

**Fig 1:**
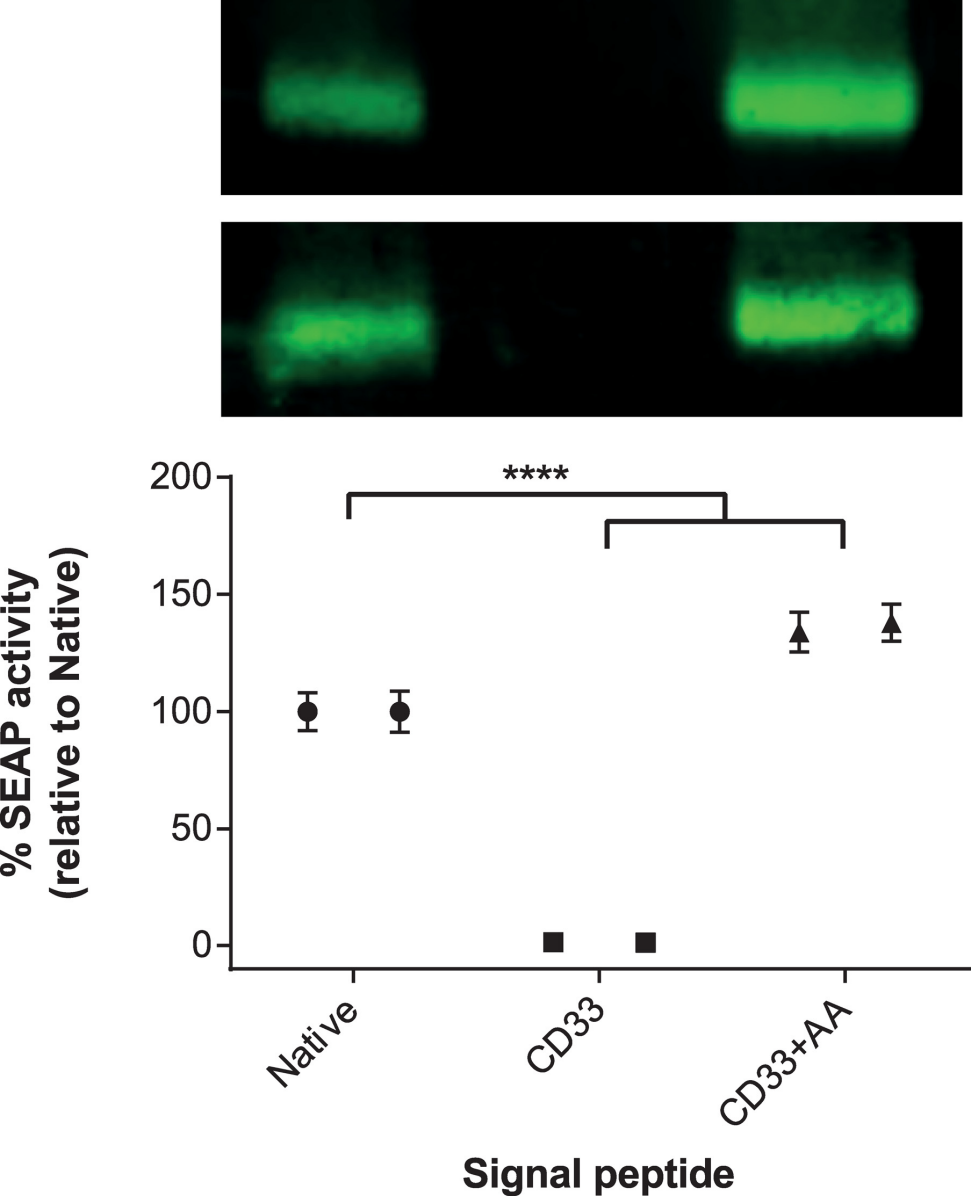
Western blot quantification of secreted SEAP levels using CD33 signal peptides. SEAP-encoding constructs were transiently expressed in HEK293 cells and the level of secreted SEAP in the conditioned media was visualised by Western blotting Full-size Western blots are available in S2 Fig. The functional activity of SEAP within the conditioned media was measured using a chemiluminescent substrate (Great EscAPe™ SEAP Chemiluminescence Kit 2.0 (Clontech, Takara)). The amount of SEAP was calculated based on linear regression analysis using a standard curve, and the percentage change compared to the control sample (Native-SEAP), was determined. Statistical analyses for each of the duplicate experiments were performed using a one-way ANOVA (Dunnett’s correction for multiple comparisons; **** = P ≤ 0.0001). Data for each sample from the duplicate experiments is shown side-by-side to highlight inter-experimental variability. Error bars represent the standard error of the mean from six replicate assay wells.

To investigate the effect of changing the signal peptide of SEAP to that of a non-native signal peptide, we first chose to investigate the commonly used human CD33 signal peptide that has been shown to improve the secretion of proteins such as IL-15 [14]. The DNA sequence encoding SEAP was codon-optimised for human expression and generated by gene synthesis. The DNA was subcloned into the expression plasmid such that an additional two alanine (A) residues were inserted between the signal peptide and the mature SEAP protein (CD33-AA). To explore the impact of the two additional residues on secreted protein levels, a plasmid that contained the CD33 signal peptide directly adjacent to the mature SEAP protein sequence was generated in parallel. Plasmids encoding C-terminally histidine-tagged SEAP with the native, CD33 and CD33-AA signal peptides were transfected into HEK293 cells and the conditioned media was collected six days later. The amount of secreted protein was determined via Western blot using an anti-his tag antibody and separately via an assay to determine SEAP activity (Fig 1). The native SEAP protein expressed well in the HEK293 cells, however, we observed that replacing the native signal peptide with that of human CD33 reduced the amount of SEAP to undetectable levels. Interestingly, however, the addition of two alanine residues between the CD33 signal peptide and the mature SEAP sequence completely restored secreted levels.

### Comparison of different signal peptides and SEAP expression levels

**Fig 2:**
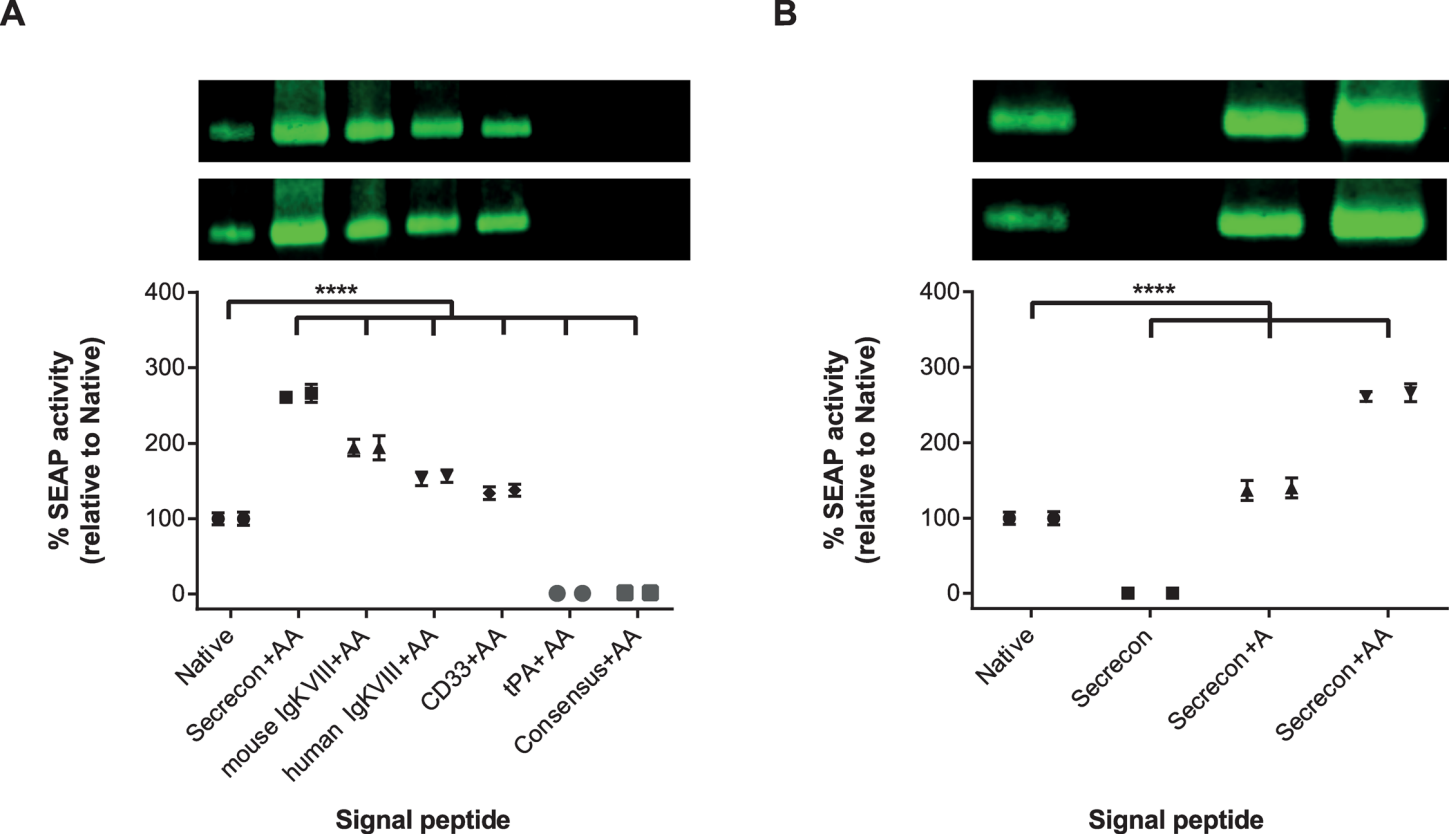
Western blot quantification of secreted SEAP levels using different signal peptides. SEAP-encoding constructs were transiently expressed in HEK293 cells and the level of secreted SEAP in the conditioned media was visualised by Western blotting. Full-size Western blots are available in S3 Fig and S5 Fig. The functional activity of SEAP within the conditioned media was measured using a chemiluminescent substrate (Great EscAPe™ SEAP Chemiluminescence Kit 2.0 (Clontech, Takara)). The amount of SEAP was calculated based on linear regression analysis using a standard curve, and the percentage change compared to the control sample (Native-SEAP), was determined. Statistical analyses for each of the duplicate experiments were performed using a one-way ANOVA (Dunnett’s correction for multiple comparisons; **** = P ≤ 0.0001). Data for each sample from the duplicate experiments is shown side-by-side to highlight inter-experimental variability. Error bars represent the standard error of the mean from six replicate assay wells.

To investigate whether this phenomenon would be observed for other signal peptides and to identify potentially superior sequences, a panel of alternative signal peptides were chosen for further analysis (Table 1). These included the signal peptides from human and mouse IgG kappa that are used routinely for the generation of antibody therapeutics [15], and tissue plasminogen activator (tPA) [16]. In addition, we included two non-native sequences; one termed “secrecon” that had been identified previously by Barash et al. [17] as scoring highly in a hidden Markov model algorithm; and one that had been generated by incorporating the most prevalent amino acids in each position of all known human 16-residue signal peptides into an artificial sequence (termed “consensus”; MLLLLLLLLLLALALA). We chose to derive a consensus sequence of a specified length of 16 amino acids to enable a comparison with the CD33 signal peptide that is also 16 amino acids in length. All five signal peptide sequences were cloned into the SEAP expression plasmid encoding two adjacent alanine residues, and versions with the signal peptide placed adjacent to the mature SEAP peptide were generated by gene synthesis. These plasmids, along with the native SEAP plasmid and the CD33-containing plasmids were transfected into HEK293 cells and the level of SEAP was determined by Western blot (S4 Fig). In agreement with the results obtained with the CD33 signal peptide, we observed a complete abrogation of detectable SEAP protein from plasmids where the non-native signal peptide had been placed adjacent to the mature peptide (S4 Fig). When the plasmids with the two additional alanine residues are used, however, SEAP expression was restored using all but two (tPA and “consensus”) non-native signal peptides (Fig 2A). Indeed, when incorporating the secrecon signal peptide in combination with two alanine residues upstream of the mature sequence, the level of secreted SEAP could be improved over two-fold, compared to native levels.

**Table 1:** Summary of the signal peptides used in this study.

Signal Peptide	Amino acid sequence	Information
Secrecon	MWWRLWWLLLLLLLLWPMVWA	Ideal human signal sequence by Barash *et*.*al* [17]
Mouse IgKVIII	METDTLLLWVLLLWVPGSTG	Mouse Ig kappa chain V-III region
Human IgKVIII	MDMRVPAQLLGLLLLWLRGARC	Human Ig kappa light chain V-III region
CD33	MPLLLLLPLLWAGALA	Human CD33
tPA	MDAMKRGLCCVLLLCGAVFVSPS	Human tissue plasminogen activator
Consensus	MLLLLLLLLLLALALA	Consensus 16aa signal peptide
Native	MLLLLLLLGLRLQLSLG	Native secreted alkaline phosphatase (SEAP)

To determine whether two alanine residues are required to enable secretion of SEAP with a heterologous signal peptide, or whether a single alanine would be sufficient, an additional expression plasmid containing a single alanine between secrecon, the high-yielding signal peptide, and SEAP was generated. The amount of secreted SEAP was determined in comparison to equivalent versions with two or no alanine residues. The quantitative data obtained (Fig 2B) suggests that the presence of a single alanine is necessary and sufficient for secretion of SEAP, although the level was two-fold lower than the two alanine-containing protein.

To verify that the processing of the secrecon signal peptide had occurred between the expected amino acids, N-terminal sequencing was performed on the purified proteins. The following N-terminal amino acids were observed, confirming that the heterologous secrecon signal peptide had been processed correctly: Native = IIPVE; secrecon-A = AIIPV; secrecon-AA = AAIIP (data not shown).

### Impact of different amino acids between the signal peptide and mature SEAP protein

**Fig 3:**
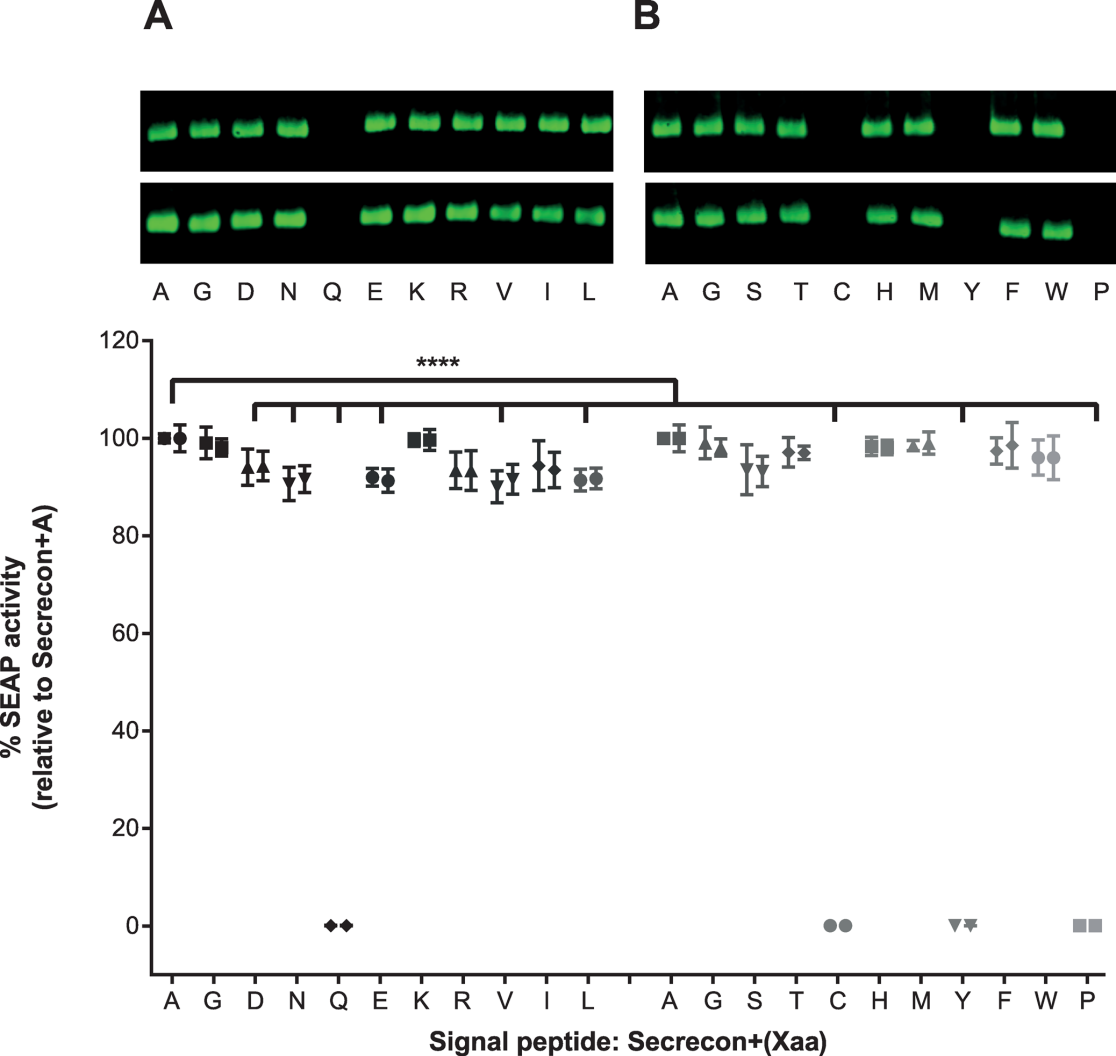
Western blot quantification of secreted SEAP levels using different +1 residues. Secrecon-SEAP-encoding constructs were transiently expressed in HEK293 cells and the level of secreted SEAP in the conditioned media was visualised by Western blotting. Panels A and B represent 2 separate Western blots representing the data from the 18 different samples. Full-size Western blots are available in S6 Fig. The functional activity of SEAP within the conditioned media was measured using a chemiluminescent substrate (Great EscAPe™ SEAP Chemiluminescence Kit 2.0 (Clontech, Takara)). The amount of SEAP was calculated based on linear regression analysis using a standard curve, and the percentage change compared to the control sample (Native-SEAP), was determined. Statistical analyses for each of the duplicate experiments were performed using a one-way ANOVA (Dunnett’s correction for multiple comparisons; **** = P ≤ 0.0001). Data for each sample from the duplicate experiments is shown side-by-side to highlight inter-experimental variability. Error bars represent the standard error of the mean from six replicate assay wells.

The experiments conducted so far have demonstrated that a signal peptide and the adjacent +1 residue need to be compatible to allow efficient recognition and processing of the signal peptide enabling SEAP to be secreted. The secrecon signal peptide in combination with a single alanine proved to be more efficient than the native SEAP signal peptide. In an attempt to identify whether the preference for certain amino acids in the +1 position was restricted to alanine, we systematically replaced alanine with all 19 alternative amino acids via gene synthesis and assessed their ability to enable secretion of SEAP using Western blot quantification and a SEAP activity assay (Fig 3). The results indicate that the majority (16/20) of amino acids are permitted at the +1 position in the context of the secrecon peptide and SEAP with only a modest reduction in SEAP levels. Interestingly, however, we observed no expression or activity when certain amino acids were used in this +1 position, namely tyrosine, cysteine, glutamine and proline.

### The expression of endogenous proteins containing “unfavourable” amino acids at +1

**Fig 4:**
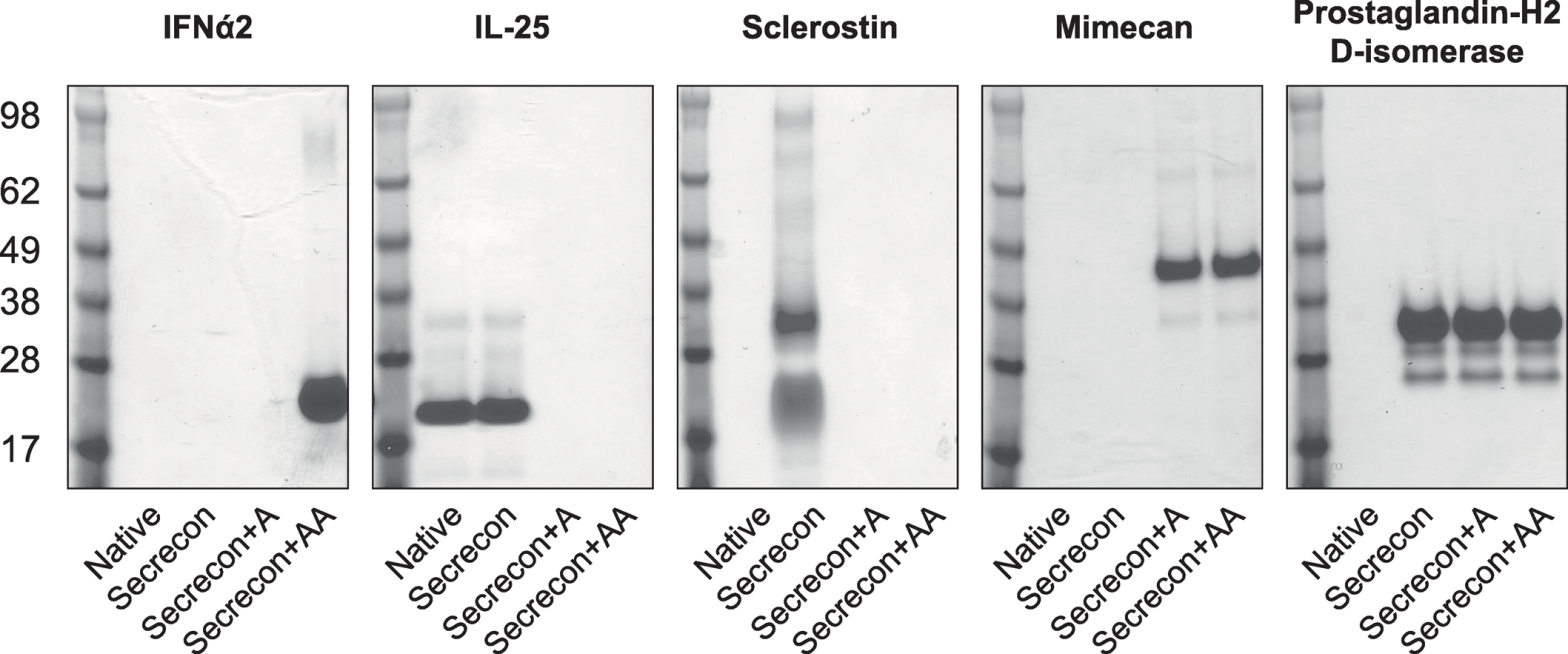
SDS-PAGE analysis of proteins with different signal peptide/adjacent amino acid combinations. SDS-PAGE gels illustrating purified secreted protein levels for different proteins with signal peptide/adjacent amino acid combinations. The samples were run alongside a two-colour Odyssey molecular weight marker for size determinationFull-size duplicate SDS-PAGE gels are available in S7 Fig.

The observation that tyrosine, cysteine and proline were not favoured at the +1 position correlates with a lower prevalence of these amino acids in the +1 position of endogenous proteins [1,2,18], which could reflect a natural mechanism to regulate the expression and secretion of such proteins to an appropriate physiological level. When it is necessary to generate such proteins recombinantly, however, this natural mechanism may need to be overcome to produce sufficient quantities of protein for downstream applications. We sought to investigate whether proteins that contain such “detrimental” amino acids in their +1 position do indeed suffer from a low expression level in mammalian cells, when they are present in the context of their native signal peptide. In parallel, we investigated the effect of replacing the natural signal peptide with secrecon, and furthermore the effect of adding one or two alanine residues in the +1 and +2 position, upstream of the mature protein. In addition to proteins with unfavourable amino acids in the +1 positions we also included another protein that encoded the more favourable alanine at position +1. The naturally secreted proteins chosen for this experiment were human interleukin 25 (IL-25; tyrosine at +1), human interferon alpha 2a (IFNα2; cysteine at +1), human sclerostin (glutamine at +1), human mimecan (proline at +1) and human prostaglandin-H2 D-isomerase (alanine at +1). Each polyhistidine-tagged protein construct was transfected into suspension HEK293 cells and the protein was purified from the conditioned media using HisTrap excel resin. The proteins were visualised by SDS-PAGE (Fig 4) and revealed good levels of homogeneity for IFNα2, IL-25 and mimecan, whereas a degree of heterogeneity was observed for sclerostin and prostaglandin-H2 D-isomerase. This level of heterogeneity is not unusual following a single step purification, and could represent different protein glycoforms. Nevertheless, the data is sufficient to identify conditions in which the proteins under investigation can be secreted. An interesting observation is that, for the different signal peptide and amino acid combinations, there seems to be an “all or nothing” phenomenon whereby the protein is either absent, or is secreted at an equivalent level when different combinations are used. Moreover, it is important to note that the effect of altering the signal peptide and adding additional residues is different for each protein. Three of the four proteins that contained undesirable +1 residues could not be expressed recombinantly in HEK293 cells with their native signal peptide, whereas replacement with secrecon +/- alanine residues enabled expression. The requirement for additional alanines was different for all three proteins, however; IFNα2 could only be produced when two alanine residues were placed between secrecon and the mature protein sequence whereas sclerostin could only tolerate the secrecon signal peptide directly adjacent to the mature protein. Surprisingly, for prostaglandin-H2 D-isomerase, which contains alanine at the +1 position and thus anticipated to be favourable, protein could not be detected when the native signal peptide was used. Only when the secrecon signal peptide was incorporated, either alone or in combination with alanine residues separating it from the mature protein, could secreted expression be detected. As IFNα2 is a clinically useful molecule, and we were able to express and purify material in this study, it was important also to demonstrate that the material purified in this study was indeed functional. Through the use of a reporter-based assay, we were indeed able to confirm the activity of purified IFNα2 (S8 Fig). We have shown here that, for a small panel of proteins, screening just four different signal peptide and alanine combinations in parallel, we have identified at least one approach that led to successful protein expression.

## Discussion

The successful production of recombinant proteins often requires the optimisation of several factors including expression conditions, gene sequence manipulation and addition of appropriate purification tags. Here we focussed on the potential of identifying a non-native signal peptide that would have universal applicability in improving the secretion of recombinant proteins, in particular those that prove refractory to expression with their native signal peptide.

In conducting these experiments we identified that, in the context of a model protein (SEAP), simply adjoining a heterologous signal peptide to a mature protein sequence does not guarantee secreted expression. On the contrary, the expression of SEAP, when placed directly adjacent to any of the non-native signal peptides tested, was completely abrogated. Restoration of SEAP expression was achieved when additional amino acids (either one or two alanines) were placed upstream of the isoleucine that represents the start of the mature SEAP sequence. This data demonstrates that the combination of the native SEAP signal peptide and its mature protein is preferred over all of the heterologous alternatives tested, and that the identity of the amino acids downstream of a signal peptide are critical for successful protein secretion.

At first glance it would appear that the lack of expression for the secrecon-SEAP protein is contradictory to that obtained by Barash et al., 2002 [17], where successful SEAP expression with the secrecon signal peptide was achieved. Upon review of the cloning strategy employed, however, it is clear that the signal peptide and mature peptide in their study are separated by two amino acids (glutamate and phenylalanine) due to the presence of an *Eco*RI restriction site. The data from Barash et al. [17], therefore correlates well with the observation in this study that by inserting two amino acids between the heterologous signal peptide and the mature SEAP sequence, expression can be restored.

Once the requirement for additional amino acids between the signal and mature sequences of SEAP had been identified, a comparison of different heterologous signal peptides, incorporating the additional alanine residues, was conducted with the ultimate aim of identifying one that could improve secreted protein levels beyond that obtained with the native sequence. Indeed, we were successful in achieving improved SEAP levels using the synthetic sequence, secrecon, further supporting the data from Barash et al. [17]. The CD33, and Igκ signal peptides provided somewhat higher SEAP levels compared to the native signal peptide, however, no protein was detected when the tPA and the 16 amino acid consensus signal sequences were used. When the alternative signal peptides, in the context of the mature SEAP sequence and spacer residues, were run through a signal peptide prediction algorithm SignalP 4.1 [19] (data not shown), the scores obtained did not correlate with the level of secreted protein observed indicating that prediction of the presence of signal peptides cannot be used to infer secreted protein levels, and that they need to be determined empirically.

As the secrecon signal peptide contains an alanine at the -1 position, the addition of further alanine residues between the secrecon peptide and SEAP could affect the recognition of the signal peptide by creating different and/or multiple potential cleavage sites, and thus generate a heterogeneous preparation consisting of proteins with different N-terminal residues. In conducting N-terminal sequence analysis, however, we were able to rule out this possibility as the protein species sequenced contained the anticipated N-termini, indicating correct signal peptide processing. It should be noted, however, that although the addition of two small neutral amino acids to the N-terminus of a protein is unlikely to have a detrimental effect on the activity or folding of a protein, and did not affect the activity of SEAP, there may be occasions where this would indeed be the case. Therefore, minimising the number of additional amino acids incorporated is preferable to obtain a recombinant protein that more closely resembles the native mature protein. Indeed, we were able to obtain high levels of SEAP from the secrecon-SEAP variant with a single alanine (secrecon-A-SEAP), in addition to the variant with two additional alanines (secrecon-AA-SEAP).

The striking difference observed in secreted levels of SEAP when an alanine is placed at the +1 position, adjacent to secrecon as opposed to the isoleucine of the mature SEAP protein, led us to question the impact of other amino acids in the +1 position. The data obtained from the systematic analysis where we replaced alanine for every other amino acid suggests that most residues are accepted at that position, but there is a clear negative impact of some amino acids on the secreted levels of SEAP. In particular tyrosine, proline, cysteine and glutamine are not tolerated in this position. Interestingly, in a study in which the N-terminus of 270 eukaryotic proteins was empirically determined by N-terminal sequencing following expression in CHO cells, the prevalence of proline and tyrosine residues in the +1 position was strikingly low [18]. In addition, it has been shown that proline is entirely absent from the +1 position in the signal peptide of prokaryotic proteins and that when a proline is artificially incorporated at the +1 position it serves as a non-cleavable competitor for the signal peptidase, reducing processing of endogenous proteins and affecting cellular function and growth [20–22]. The low natural occurrence of proline in eukaryotic proteins may suggest that a similar mechanism is operating in eukaryotic cells to prevent appropriate signal peptide recognition and/or cleavage and subsequent secretion from cells, although this has yet to be demonstrated. Based on the Zhang et al. study [18] the prevalence of glutamine residues in the +1 position was high (approx. 10%), however, the presence of glutamine in +1 position in the context of secrecon and SEAP was detrimental to secreted protein levels, implying that the prevalence of certain amino acids in this small dataset cannot be used to predict successful secreted protein outcomes.

When a representative set of proteins containing the four “detrimental” amino acids or alanine at the +1 position was expressed in various signal peptide/spacer combinations, the results obtained were difficult to interpret in a systematic way. Three of the four proteins with undesirable +1 amino acids indeed did not express well in HEK293 cells, but one, IL-25 (tyrosine at +1), did. In addition, the protein with a naturally occurring alanine residue at +1 (Prostaglandin-H2 D-isomerase) did not express when its native signal peptide was used, but did when the secrecon signal peptide was used either alone or in combination with spacer residues. For the three proteins that were not successfully detected when the native signal peptide was used, one or more of the three alternative secrecon-based strategies did yield secreted protein. For mimecan that encodes a proline at the +1 position, no secreted protein was detected with the native signal peptide, as was also the case when proline was inserted at the +1 position for secrecon-SEAP. Furthermore, no secretion was observed when the signal peptide was replaced with the secrecon sequence. When one or two alanine residues were placed between secrecon and the mature peptide sequence, secreted protein expression was achieved—a result that correlates well with the analysis performed by Zhang et al [18] showing that proline residues are not preferred at the +1 position, but are favoured at the +2 position and beyond. Another protein examined in this study, IFNα2 is used for the treatment of viral infection and cancer. Human recombinant IFNα2 used in the clinic is synthesized in bacterial systems, and yields of a few grams (3 to 5) of recombinant human IFNα2 per litre of culture can be produced [23]. Bacterially produced recombinant human IFNα2, however, is misfolded and requires refolding into its native conformation; a process that typically yields a lower than 20% recovery rate [23] and often results in loss of activity. Furthermore, bacterially produced recombinant human IFNα2 lacks the naturally occurring O-glycosylation and this non-glycosylated form of human recombinant IFNα2 has a lower activity and shorter serum half-life than mammalian-derived glycosylated forms [24, 25]. Expression of IFNα2 in mammalian systems could, therefore, be a viable alternative, however yields are typically low, and this could at least in part be due to the presence of a signal peptide and mature peptide combination that is not optimal for high level expression in heterologous systems. The strategy adopted in this study involving the use of a heterologous signal peptide in combination with spacer amino acids to successfully express active recombinant IFNα2 in HEK293 cells, could provide significant benefits in the production of this important therapeutic protein.

In conducting this study we have confirmed findings previously reported that the secreted level of proteins can be significantly influenced by replacing the signal peptide sequence with that of a heterologous protein, or with a synthetic signal peptide. Moreover, we have demonstrated that amino acids present in the mature peptide play a crucial role in the determining whether a protein is secreted from a cell. Whether this effect is purely a post-translational one involving recognition by the SRP receptor or cleavage by the signal peptidase, or involves transcription and translation rates was not the subject of this study, but warrants further attention. From the results presented here it is difficult to make any overarching conclusions and recommendations based on the use of a universal signal peptide and/or additional residue combination as the impact of such modifications appears to be context specific. A systematic study encompassing a larger panel of proteins and signal peptide sequences is required to be able to identify any rules and requirements for particular primary sequence features, as well as size, charge and secondary structure considerations for successful secreted protein production. The availability of crystal structures for the key components of the signal peptide recognition and cleavage pathways would also enable predictive modelling studies to be performed, that could drive a more rational design of optimal signal peptide and mature protein combinations and avoid the need for additional residues.

In conclusion, we have reinforced the importance of the mature protein sequence in combination with the signal peptide for achieving recombinant protein expression. Our data, with a small panel of secreted proteins, suggests that it will be challenging to predict whether or not a protein will express recombinantly in mammalian cell culture solely by taking into account the +1 amino acid, and that replacing the signal peptide with a “universal” signal peptide will not overcome expression issues in every case. Nevertheless, by employing the secrecon signal peptide and generating a limited number of alternative expression plasmids, the successful expression of four different recombinant proteins from mammalian cells that could not be obtained with their native sequence was achieved. It is anticipated that this approach could be used to successfully generate proteins that are of significant biological and clinical value.

## Supporting Information

S1 Fig Sequence logo depicting the frequency of amino acids in a 16 amino acid long signal peptide. The frequency of residues was analysed from an alignment of 24 human 16 amino acid long signal peptide sequences to generate a “consensus” sequence signal peptide. The height of each residue represents the frequency of that amino acid in the 24 signal peptides analysed. The figure was generated using the WebLogo tool (http://weblogo.berkeley.edu/logo.cgi).
(PDF)

S2 Fig Full-size Western blots illustrating secreted SEAP levels using native and CD33 signal peptides. Panels **A** and **B** represent duplicate Western blots for Fig 1 (**M** = two-colour Odyssey molecular weight marker). Blots correspond to Fig 1.
(PDF)

S3 Fig Full-size Western blots illustrating secreted SEAP levels using different signal peptides. Panels **A** and **B** represent duplicate Western blots for Fig 3 (**M** = two-colour Odyssey molecular weight marker). Blots correspond to Fig 2A.
(PDF)

S4 Fig Full-size Western blots illustrating secreted SEAP levels using different signal peptides +/- two adjacent alanines (M = two-colour Odyssey molecular weight marker). (PDF)

S5 Fig Full-size Western blots illustrating secreted SEAP levels using secrecon signal peptides. Panels **A** and **B** represent duplicate Western blots for Fig 4 (**M** = two-colour Odyssey molecular weight marker). Blots correspond to Fig 2B.
(PDF)

S6 Fig Full-size Western blots illustrating secreted SEAP levels using different signal peptide/adjacent amino acid combinations. Panels **A** and **B** represent duplicate Western blots for Fig 3A; panels **C** and **D** represent duplicate Western blots for Fig 3B (**M** = two-colour Odyssey molecular weight marker). Blots correspond to Fig 3.
(PDF)

S7 Fig Duplicate full-size SDS-PAGE gels illustrating purified secreted protein levels for different proteins with signal peptide/adjacent amino acid combinations (M = two-colour Odyssey molecular weight marker). Blots correspond to Fig 4.
(PDF)

S8 Fig Functional activity of human IFNα2. iLite^TM^ Type I IFN Assay Ready cells (EuroDiagnostica) were incubated with increasing concentrations of purified IFNα2 for 18 hours and the luminescence was measured following addition of a luciferase substrate (Bright-Glo^TM^, Promega). Error bars represent the 5–95 percentiles of the mean from four replicate assay wells. The concentration of IFNα2 required for a half-maximal (EC50) response was determined using non-linear regression analysis (log [agonist] vs. response, 3-parameter fit curve) in GraphPad Prism (San Diego, CA).
(PDF)
