# Supplementary figures and images for: Overcoming the Refractory Expression of Secreted Recombinant Proteins in Mammalian Cells through Modification of the Signal Peptide and Adjacent Amino Acids
Source: PLoS One. 2016 May 19;11(5):e0155340. doi: 10.1371/journal.pone.0155340 (PMC4873207; doi:10.1371/journal.pone.0155340)

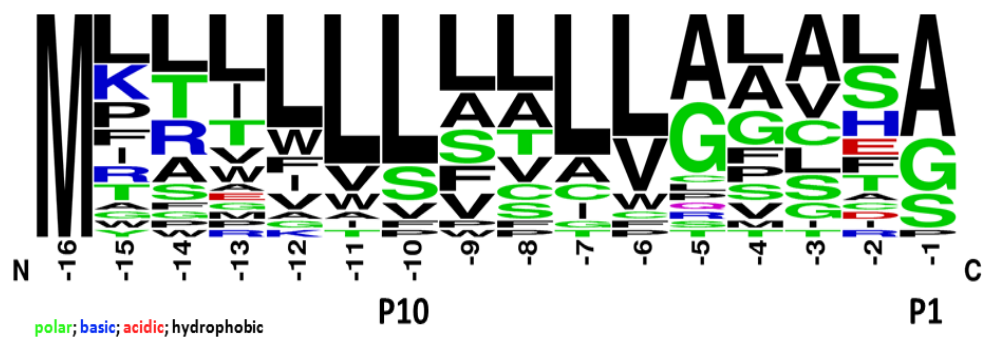

S1 Fig. Sequence logo depicting the frequency of amino acids in a 16 amino acid long signal peptide.

Supplement: S1 Fig — The frequency of residues was analysed from an alignment of 24 human 16 amino acid long signal peptide sequences to generate a “consensus” sequence signal peptide. The height of each residue represents the frequency of that amino acid in the 24 signal peptides analysed. The figure was generated using the WebLogo tool (http://weblogo.berkeley.edu/logo.cgi). (PDF) [file pone.0155340.s001.pdf]

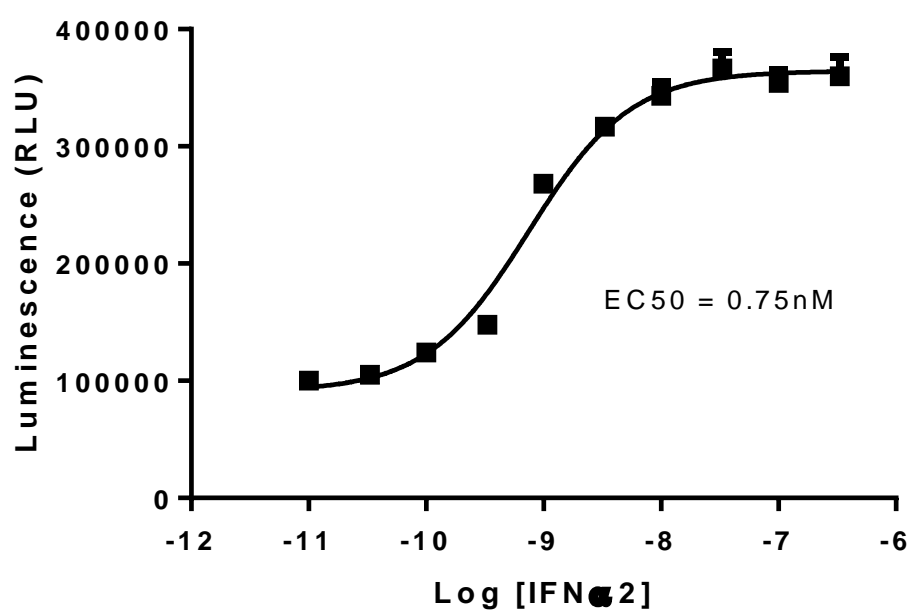

S8 Fig. Functional activity of human IFN $\alpha$ 2.

Supplement: S8 Fig — iLiteTM Type I IFN Assay Ready cells (EuroDiagnostica) were incubated with increasing concentrations of purified IFNα2 for 18 hours and the luminescence was measured following addition of a luciferase substrate (Bright-GloTM, Promega). Error bars represent the 5–95 percentiles of the mean from four replicate assay wells. The concentration of IFNα2 required for a half-maximal (EC50) response was determined using non-linear regression analysis (log [agonist] vs. response, 3-parameter fit curve) in GraphPad Prism (San Diego, CA). (PDF) [file pone.0155340.s008.pdf]
