# Supplementary material for: Overcoming the Refractory Expression of Secreted Recombinant Proteins in Mammalian Cells through Modification of the Signal Peptide and Adjacent Amino Acids
Source: PLoS One. 2016 May 19;11(5):e0155340. doi: 10.1371/journal.pone.0155340 (PMC4873207; doi:10.1371/journal.pone.0155340)
Supplement: S4 Fig — (PDF) [file pone.0155340.s004.pdf]

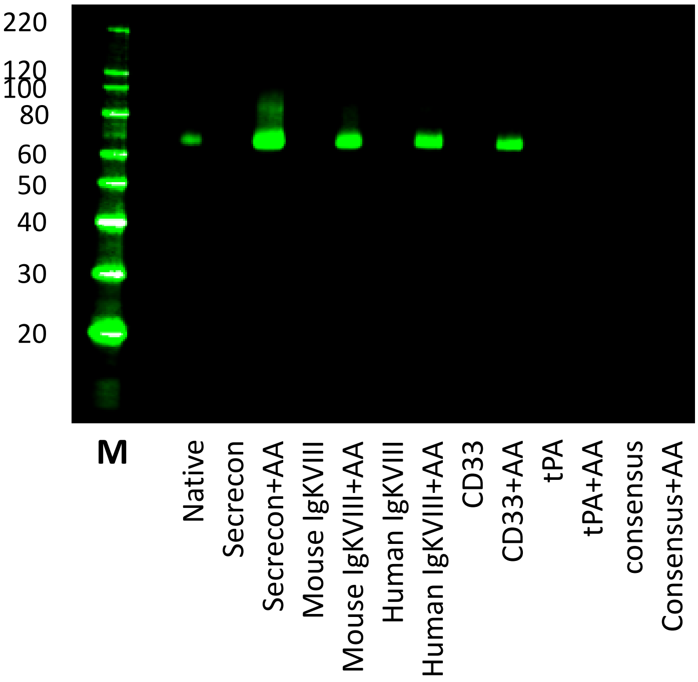

S4 Fig: Full-size Western blots illustrating secreted SEAP levels using different signal peptides +/- two adjacent alanines
