# Supplementary material for: Overcoming the Refractory Expression of Secreted Recombinant Proteins in Mammalian Cells through Modification of the Signal Peptide and Adjacent Amino Acids
Source: PLoS One. 2016 May 19;11(5):e0155340. doi: 10.1371/journal.pone.0155340 (PMC4873207; doi:10.1371/journal.pone.0155340)
Supplement: S5 Fig — Panels A and B represent duplicate Western blots for Fig 4 (M = two-colour Odyssey molecular weight marker). Blots correspond to Fig 2B. (PDF) [file pone.0155340.s005.pdf]

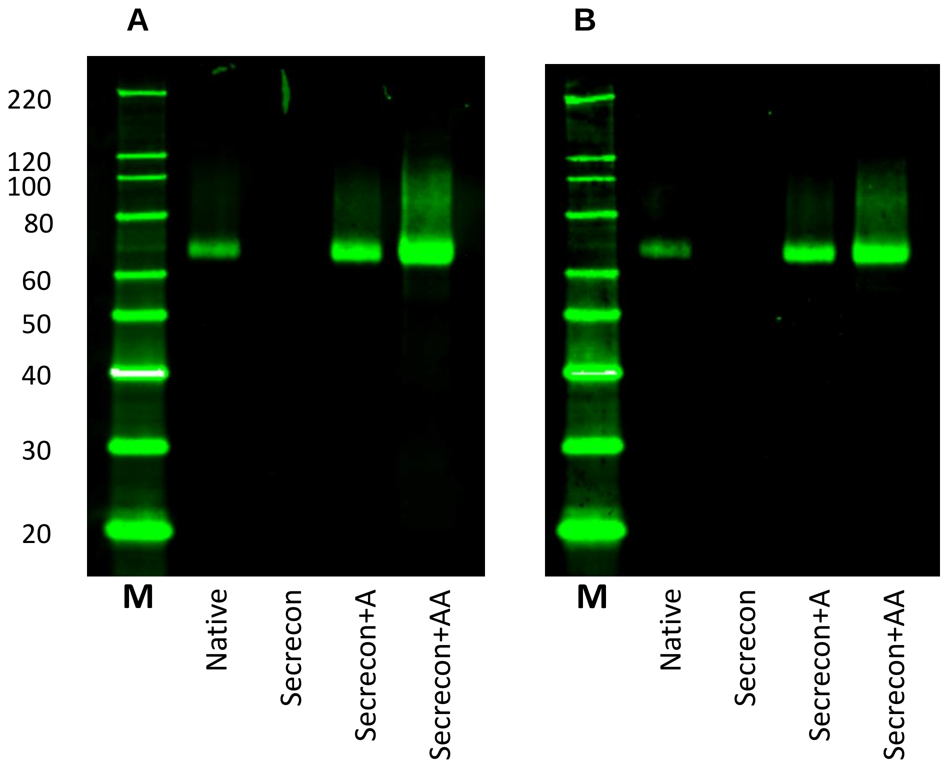

S5 Fig. Full-size Western blots illustrating secreted SEAP levels using secrecon signal peptides.
