# Supplementary material for: Overcoming the Refractory Expression of Secreted Recombinant Proteins in Mammalian Cells through Modification of the Signal Peptide and Adjacent Amino Acids
Source: PLoS One. 2016 May 19;11(5):e0155340. doi: 10.1371/journal.pone.0155340 (PMC4873207; doi:10.1371/journal.pone.0155340)
Supplement: S7 Fig — Blots correspond to Fig 4. (PDF) [file pone.0155340.s007.pdf]

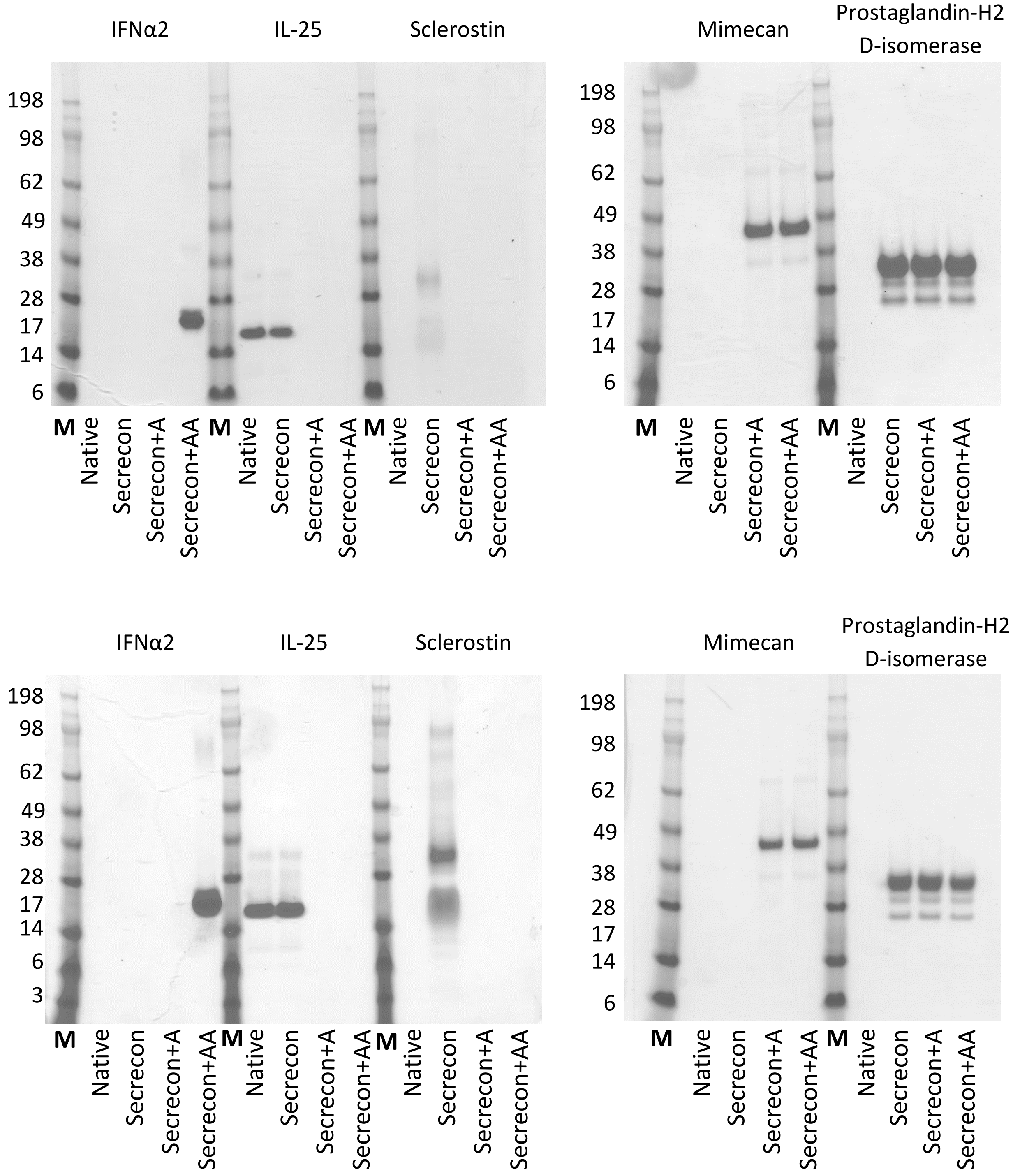

S7 Fig. Duplicate full-size SDS-PAGE gels illustrating purified secreted protein levels for different proteins with signal peptide/adjacent amino acid combinations
